# Novel Insight Into the Natural History of Short QT Syndrome

**DOI:** 10.1016/j.jacc.2013.09.078

**Published:** 2014-04-08

**Authors:** Andrea Mazzanti, Ajita Kanthan, Nicola Monteforte, Mirella Memmi, Raffaella Bloise, Valeria Novelli, Carlotta Miceli, Sean O'Rourke, Gianluca Borio, Agnieszka Zienciuk-Krajka, Antonio Curcio, Andreea Elena Surducan, Mario Colombo, Carlo Napolitano, Silvia G. Priori

**Affiliations:** ∗ Molecular Cardiology, IRCCS Salvatore Maugeri Foundation, Pavia, Italy; † Cardiovascular Genetics Program, Leon H. Charney Division of Cardiology, New York University School of Medicine, New York, New York; ‡ Department of Cardiology and Electrotherapy, Medical University of Gdansk, Gdansk, Poland; § Smartech, Milan, Italy; ‖ Department of Molecular Medicine, University of Pavia, Pavia, Italy

**Keywords:** genetics, short QT syndrome, sudden cardiac death, ventricular arrhythmias, ArrS, arrhythmic storm, BrS, Brugada syndrome, CA, cardiac arrest, CI, coupling interval, ECG, electrocardiogram, ICD, implantable cardioverter-defibrillator, IQR, interquartile range, PES, programmed electrical stimulation, QTc, corrected QT, SQTS, short QT syndrome, VEB, ventricular ectopic beat, VF, ventricular fibrillation, VT, ventricular tachycardia

## Abstract

**Objectives:**

This study intends to gain further insights into the natural history, the yield of familial and genetic screening, and the arrhythmogenic mechanisms in the largest cohort of short QT syndrome (SQTS) patients described so far.

**Background:**

SQTS is a rare genetic disorder associated with life-threatening arrhythmias, and its natural history is incompletely ascertained.

**Methods:**

Seventy-three SQTS patients (84% male; age, 26 ± 15 years; corrected QT interval, 329 ± 22 ms) were studied, and 62 were followed for 60 ± 41 months (median, 56 months).

**Results:**

Cardiac arrest (CA) was the most frequent presenting symptom (40% of probands; range, <1 month to 41 years). The rate of CA was 4% in the first year of life and 1.3% per year between 20 and 40 years; the probability of a first occurrence of CA by 40 years of age was 41%. Despite the male predominance, female patients had a risk profile superimposable to that of men (p = 0.49). The yield of genetic screening was low (14%), despite familial disease being present in 44% of kindreds. A history of CA was the only predictor of recurrences at follow-up (p < 0.0000001). Two patterns of onset of ventricular fibrillation were observed and were reproducible in patients with multiple occurrences of CA. Arrhythmias occurred mainly at rest.

**Conclusions:**

SQTS is highly lethal; CA is often the first manifestation of the disease with a peak incidence in the first year of life. Survivors of CA have a high CA recurrence rate; therefore, implantation of a defibrillator is strongly recommended in this group of patients.

Thirteen years have elapsed since the description of short QT syndrome (SQTS), a rare familial disorder characterized by an abnormally shortened cardiac repolarization and a propensity for cardiac arrest (CA) 1, 2, 3. Shortly after its discovery, mutations in genes encoding potassium and l-type cardiac calcium channels have been identified that explain the disease in a small proportion of SQTS patients (4).

To date, approximately 100 SQTS patients have been reported in the published data, and the largest review collected 61 subjects 5, 6, 7; due to the small number of cases identified, the natural history of the disease is incompletely understood, and uncertainties exist about all aspects of SQTS, from diagnosis to risk stratification and management.

Diagnostic criteria for SQTS are debated and the cutoff value of the QT interval to suspect/diagnose the disease is not established: there is a gray area for the corrected QT (QTc) interval between 370 and 330 ms, for which different authors have selected their preferred limit (6). A diagnostic score for SQTS has been proposed (6), and recently a modified version has been reported, suggesting that this may also provide prognostic indications (7). Neither criterion, however, has been validated in independent populations, and their efficacy in identifying the subjects at risk of life-threatening events remains unproven.

Because registries are a valuable approach to advance medical knowledge of rare diseases, in 2003, we added to our inherited arrhythmias database a section for collecting SQTS cases. Here we present for the first time data pertaining to 73 SQTS patients identified in 47 families.

## Methods

### Definitions

SQTS was defined as a QTc interval ≤340 ms or QTc interval between 341 ms and 360 ms and 1 or more of the following: history of CA or syncope, a family history of unexplained CA at a young age (40 years of age or younger), or a family history of SQTS. Syncope was defined as a transient loss of consciousness in the absence of alternative explanations (e.g., labyrinthitis, orthostatic hypotension, or clearly vagally mediated events). Arrhythmic storm (ArrS) was defined as ≥2 separate episodes of ventricular fibrillation/ventricular tachycardia (VF/VT) within 24 h requiring resuscitation/defibrillation.

### Study population and clinical evaluation

Of the 63 patients referred to us as “suspected SQTS” on the basis of the presence of a QTc interval ≤360 ms, 47 satisfied criteria for enrollment in this study. Four were victims of sudden death with a negative autopsy and an electrocardiogram (ECG) confirming the presence of a short QT interval. Cascade screening of family members identified 26 clinically affected relatives. Additionally, 18 young victims of sudden death without an ECG were identified in the 47 families, but they were not included in the main study given the uncertainty about the cause of death. However, because excluding these victims of sudden death may underestimate the severity of the disease, we present the analysis of the natural history of our cohort both including and excluding these patients.

In all patients, a 12-lead ECG was obtained at a stable heart rate close to 60 beats/min during daylight hours to limit the confounding effect of diurnal variability of the QT interval (8). The following electrocardiographic parameters (paper speed of 25 mm/s and 200% magnification) were measured: PR interval, RR interval, QT interval/QTc interval, QRS interval, and J point to T wave peak (J-Tpeak) interval. The QT interval was measured using the tangent method in the precordial lead presenting the highest T-wave amplitude (either V_2_ or V_3_ in 74% of cases) (8). The QTc interval was calculated using Bazett's formula.

We calculated the modified SQTS score (7) for 34 probands for whom all the parameters needed were available. To limit potential bias, we repeated the analysis for all 40 probands with follow-up, allocating maximal scores for missing information.

### Genetic screening

DNA sequencing of the open reading frame of the *KCNH2*, *KCNQ1*, and *KCNJ2* genes was performed in 42 probands and in 3 affected relatives of 3 deceased probands. Screening of *CACNA1C* and *CACNB2* genes was performed after the link between mutations in the 2 genes was reported in the published data (9). At that time, DNA suitable for genotyping was available for 35 of the 45 previously-mentioned subjects.

Genetic analysis was performed in the laboratory of the Maugeri Foundation; 1 sample was genotyped by Familion, PGxHealth, Transgenomic, Inc. (Omaha, Nebraska).

The allelic frequency in the general population of the variants identified was determined using the Exome Variant Server database (10): a minor allele frequency ≥0.04 was used as a cutoff to distinguish mutations from variants of unknown significance (11). *CACNA2D1* was not screened because the only variant reported has a minor allele frequency of 0.1% in Caucasians (12); we do not consider this a pathogenic DNA change, according to our genetic counseling rules.

For novel mutations or mutations with uncertain functional significance, we performed in vitro characterization using patch clamp techniques.

### Follow-up

Our study population included 47 probands and their 26 family members, totaling 73 SQTS patients. Four were victims of sudden death, and 7 were lost at follow-up; the remaining 62 patients were followed for 60 ± 41 months (median, 56 months; interquartile range, 43 months).

### Statistical analysis

Data are expressed as percent, mean ± SD, or median with interquartile range (IQR). Univariate analyses used the chi-square or Fisher exact test for categorical data, and the *t* test or 1-way analysis of variance for continuous variables; for skewed distributions (follow-up durations), a Mann-Whitney test was used. CA-free survival was determined applying Kaplan-Meier analysis; log-rank tests were used for significance. Significance was defined as p < 0.05.

## Results

### Demographic and clinical profiles of the study population

Demographic and electrocardiographic characteristics of the study population are shown in Table 1. The reasons for referral of the 47 probands included CA (n = 19), syncope (n = 9), family history of CA (n = 2), or the incidental finding of a short QTc interval (n = 17). Twelve probands (26%) had a family history of sudden death in young subjects; 4 had multiple sudden death victims (2.5 ± 0.6) in their family.

**Table 1:** Demographic and ECG Parameters of Study Cohort

	Probands (n = 47)	Affected Relatives (n = 26)	Total (N = 73)	p Value∗
Male	42 (89)	19 (73)	61 (84)	0.101
Age at enrollment, yrs	23 ± 11	33 ± 19	26 ± 15	0.015
RR interval, ms	941 ± 125	935 ± 94	939 ± 115	0.853
QT interval, ms	317 ± 31	321 ± 29	319 ± 30	0.626
QTc interval, ms	328 ± 23	332 ± 20	329 ± 22	0.481

Values are n (%) or mean ± SD.

QTc = corrected QT (interval).

∗ p values are for comparisons between probands and affected relatives.

Cascade screening was accepted by 36 of 47 families: 1 or more affected relatives were identified in 16 families (44%). Overall, 26 of 54 screened relatives (48%) satisfied our inclusion criteria.

Thirty-nine of 73 patients (53%) had symptoms prompting medical attention: CA (n = 19), syncope (n = 11), palpitations (n = 7), and atypical chest pain (n = 2).

**Figure 1:**
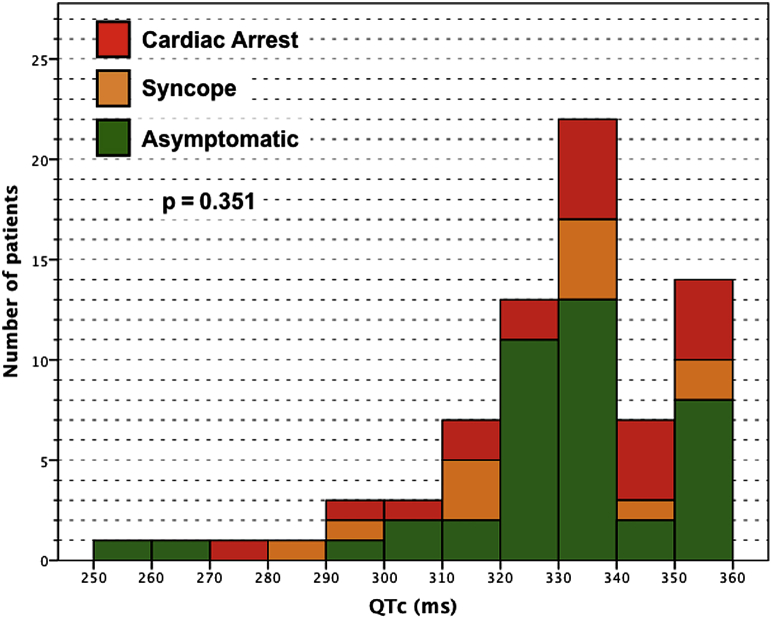
Patients Grouped According to QTc Interval and the Most Severe Symptom Experienced Each bar represents the number of patients with QTc values falling into each 10-ms interval (lower number inclusive). There was no significant correlation between QTc interval and symptoms (n = 73, p = 0.35). QTc = corrected QT.

No differences in age, sex, or electrocardiographic parameters were found among patients with different clinical manifestations (Fig. 1, Table 2).

**Table 2:** Comparison of Demographic and Electrocardiographic Parameters on the Basis of Clinical Manifestations (Considering the Severest Event in Life)

	Asymptomatic (n = 41)	Syncope (n = 12)	CA (n = 20)	p Value∗
Male	32 (78)	11 (92)	18 (90)	0.353
Age at event, yrs	—	21 ± 11	25 ± 13	0.453
RR interval, ms	948 ± 93	938 ± 153	920 ± 132	0.682
QT interval, ms	321 ± 30	314 ± 29	317 ± 32	0.765
QTc interval, ms	330 ± 22	326 ± 24	331 ± 22	0.802

Values are mean ± SD or n (%).

CA = cardiac arrest; QTc = corrected QT (interval).

∗ p values are for comparisons of all 3 groups together.

**Figure 2:**
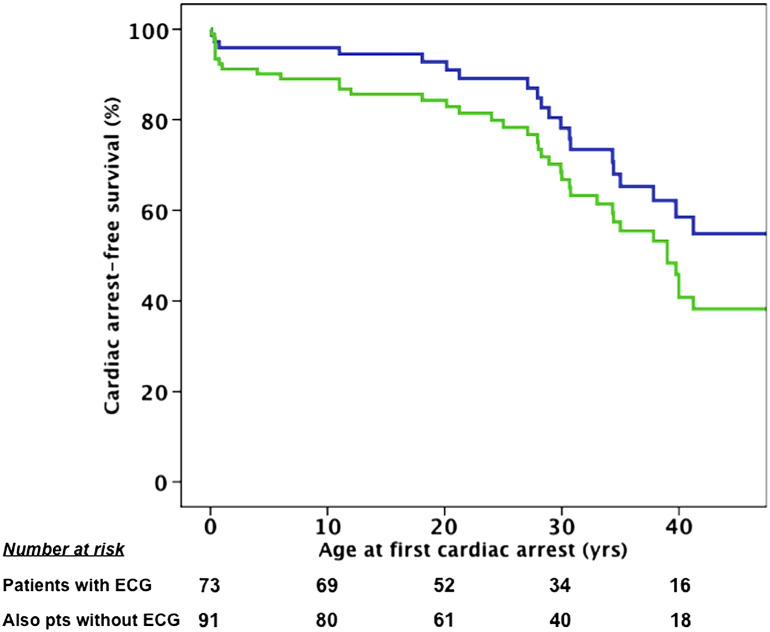
Cardiac Arrest-Free Survival Kaplan-Meier analysis: **blue line** represents main study cohort (n = 73); **green line** represents main study cohort plus the 18 young victims of sudden death without an electrocardiogram (ECG) (N = 91). pts = patients.

We observed that 20 of 73 patients (27%) experienced at least 1 occurrence of CA, 15% of which occurred within the first year of life. The cumulative probability of experiencing a first occurrence of CA by 40 years of age was 41%. We also analyzed the enlarged population, including the 18 young victims of sudden death without an ECG identified in the families; here, 38 of 91 patients (42%) experienced CA, increasing the cumulative probability of the occurrence of CA to 54%. Overall, the probability of experiencing CA in SQTS between birth and 40 years of age is estimated at between 40% and 50% (Fig. 2).

### Genetic screening

**Figure 3:**
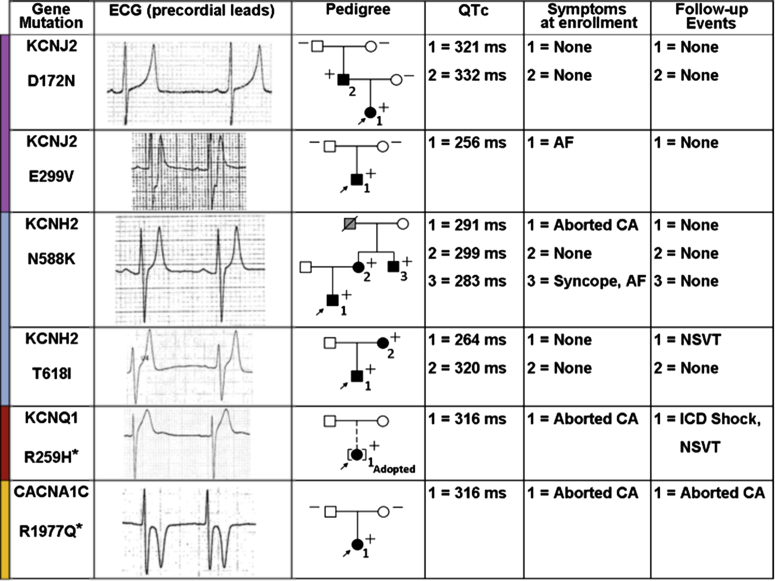
Characteristics of Genotype-Positive Families Each row refers to a kindred. Columns from left to right show gene and mutation identified; ECG of probands; the family tree; QTc interval duration; symptoms at enrollment, and events at follow-up for each mutation carrier, as numbered in the family tree. In the pedigree column, affected subjects are indicated by the solid symbols, unaffected subjects by open symbols, and sudden death victims by the grey symbols. + = mutation carrier; − = mutation noncarrier; → = probands; ☐ = male patients; ◯ = female patients. The asterisk indicates a novel mutation. AF = atrial fibrillation; CA = cardiac arrest; ECG = electrocardiogram; ICD = implantable cardioverter-defibrillator; NSVT = nonsustained ventricular tachycardia; QTc = corrected QT interval.

A pathogenic mutation in potassium channels was identified in 5 of 45 patients (11%): 2 (4.4%) in the *KCNJ2* gene (D172N, E299V), 2 (4.4%) in *KCNH2* (N588K, T618I), and 1 (2.2%) in *KCNQ1* (R259H). One patient (1/35, 2.9%) carried a *CACNA1C* mutation (R1977Q) (Fig. 3). The R259H and R1977Q variants were identified for the first time in SQTS patients, and their functional effect was confirmed by in vitro characterization (data not shown). All other mutations had been previously studied 13, 14.

Segregation studies found that 3 mutations were inherited, whereas 2, being absent in both parents, were considered likely de novo. The last genotype-positive patient had been adopted, and no information was available on her biological parents.

Interestingly, the penetrance of mutations, defined by a QTc interval ≤360 ms, was 100% in the pedigrees with multiple carriers (n = 3 probands and n = 4 family members).

Electrocardiographic data from 61 patients with complete genetic screening showed significantly shorter QTc intervals in mutation carriers versus noncarriers (p = 0.002) (Table 3). However, no difference was observed in the occurrence of CA in SQTS patients with a likely pathogenic mutation and those without (p = 0.496) (Online Fig. 1).

**Table 3:** Comparison of Demographic and Electrocardiographic Parameters on the Basis of Genotype Status

	Mutation Identified (n = 10)	No Mutation Identified (n = 51)	p Value
Male	5 (50)	46 (91)	0.007
Age at enrollment, yrs	21 ± 15	27 ± 16	0.287
PR interval, ms	151 ± 23	144 ± 22	0.378
RR interval, ms	866 ± 146	943 ± 106	0.143
QT, ms	279 ± 41	325 ± 24	0.006
QTc, ms	300 ± 26	335 ± 17	0.002
QRS, ms	80 ± 12	82 ± 11	0.639
J-T peak, ms	128 ± 46	170 ± 27	0.029

Values are n (%) or mean ± SD.

J-Tpeak = J point to T wave peak; QTc = corrected QT (interval).

### Follow-up

At follow-up, an implantable cardioverter-defibrillator (ICD) was implanted in 16 of 62 patients (single-chamber in 12 and dual-chamber in 4): 11 patients received an ICD after surviving CA, 3 because of a family history of CA and 2 because of an extremely short QTc interval (<300 ms). Four CA survivors did not receive an ICD: 1 had irreversible anoxic brain injury and 3 refused implantation.

During 60 ± 41 months of follow-up (median, 56 [IQR: 36 to 79] months), 10 of 62 patients (16%) experienced at least 1 occurrence of CA (3.2% per year) (Online Fig. 2). Nine of them had already survived an occurrence of CA: 7 had an ICD and were appropriately shocked, 1 was resuscitated by an external defibrillator, and 1 who had refused an ICD died.

**Figure 4:**
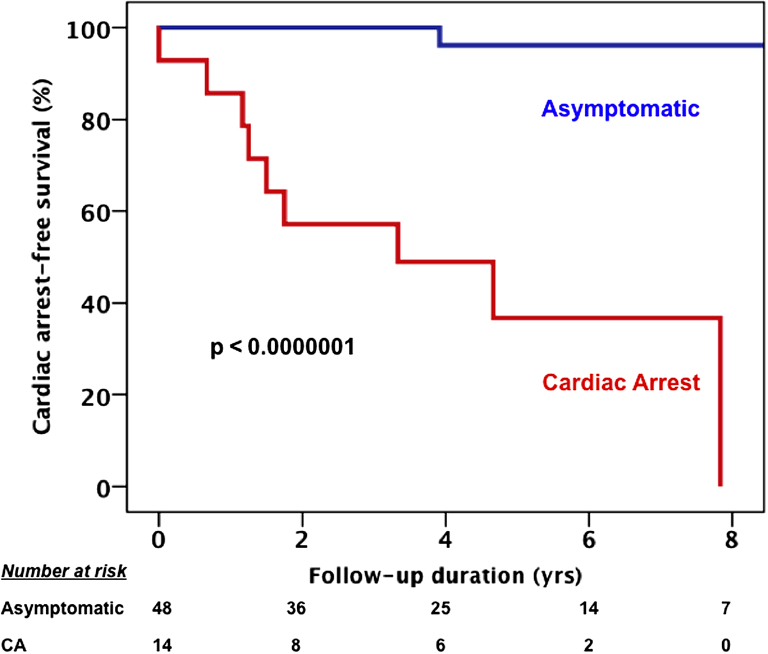
CA-Free Survival at Follow-up by CA Occurrence Before Enrollment Kaplan-Meier analysis: **red and blue lines,** respectively, represent patients with (n = 14) and without (n = 48) cardiac arrest (CA) before enrollment. Patients with CA before enrollment were more likely to experience CA during follow-up (p < 0.0000001).

The annual CA rate at follow-up was 10.6% among patients who had already experienced CA and 0.4% in those without a history of CA before the diagnosis (Fig. 4). The median follow-up in the 2 groups was not significantly different (median, 65 [IQR: 47 to 99] months vs. 53 [IQR: 21 to 76] months; p = 0.106).

ICD-related complications occurred in 5 of 16 patients and included a shock during atrial fibrillation with a rapid ventricular response (n = 1), a shock for sinus tachycardia (n = 1), infection requiring device extraction (n = 2), and psychological distress (n = 1).

### Triggers and risk factors for CA

Twenty patients experienced CA, of whom 10 had multiple events. A total of 33 separate occurrences of CA were counted: 25 occurred during rest, sleep, or quiet routine activities (20 at rest/during sleep, 3 while eating, and 2 while driving); 3 during emotional stress; and 2 during effort. The remaining 3 occurrences of CA were in infants under uncertain circumstances. Nine of 20 patients (45%) who experienced CA had an ArrS, and 3 had an ArrS recurrence during follow-up. Six of 9 patients had ≥3 VF/VT episodes per storm.

Patient-specific triggers for CA were found in 7 patients with recurrent episodes; CA occurred reproducibly under resting conditions or during sleep in 5 patients, in 1 patient while eating; and in 1 patient during emotional stress.

Univariate analysis was used to identify predictors of CA. Assessed parameters included sex, QT and QTc intervals, history of syncope, familial versus sporadic disease, family history of CA, mutation-positive status, arrhythmia induction with programmed electrical stimulation (PES), and a history of resuscitated CA. Only the last parameter was associated with an increased risk of CA.

Patients resuscitated from a previous CA had a significantly greater rate of recurrence of life-threatening arrhythmias during follow-up (9 of 14 patients) compared with patients who had never experienced CA (1 of 48 patients; log-rank test, p < 0.0000001; hazard ratio: 37.5; 95% confidence interval: 4.7 to 297.7; p = 0.01) (Fig. 4).

PES was performed in 20 patients with follow-up: VF was induced in 4 of 20 patients, and only 1 (25%) of them experienced CA, whereas 6 of 16 (38%) noninducible patients experienced CA (p = 1.0; no difference in follow-up duration, p = 0.38).

We assessed the performance of the recently proposed prognostic SQTS score (7) in 34 probands for whom we had complete information: 21 patients had a score of ≤3, corresponding to a “good prognosis.” The remaining 13 patients had a score of ≥4, indicating an adverse prognosis. Over a median follow-up of 56 (IQR: 32 to 83) months, 8 patients experienced CA, 5 of them had a score of ≤3. The incidence of CA was 4.6% per year in the good prognosis group and 4.2% per year in the adverse prognosis group (p = 0.888) during a median follow-up of 55 (IQR: 31 to 77) months versus 58 (IQR: 25 to 113) months (p = 0.889) (Online Fig. 3). When patients with incomplete scoring data were also considered, the incidence of CA remained similar (5.3% and 4.2% per year in the good and bad prognosis groups; p = 0.734) during a median follow-up of 55 (IQR: 19 to 76) months versus 67 (IQR: 31 to 96) months (p = 0.443).

### Extrasystolic beats initiating VF/VT

**Figure 5:**
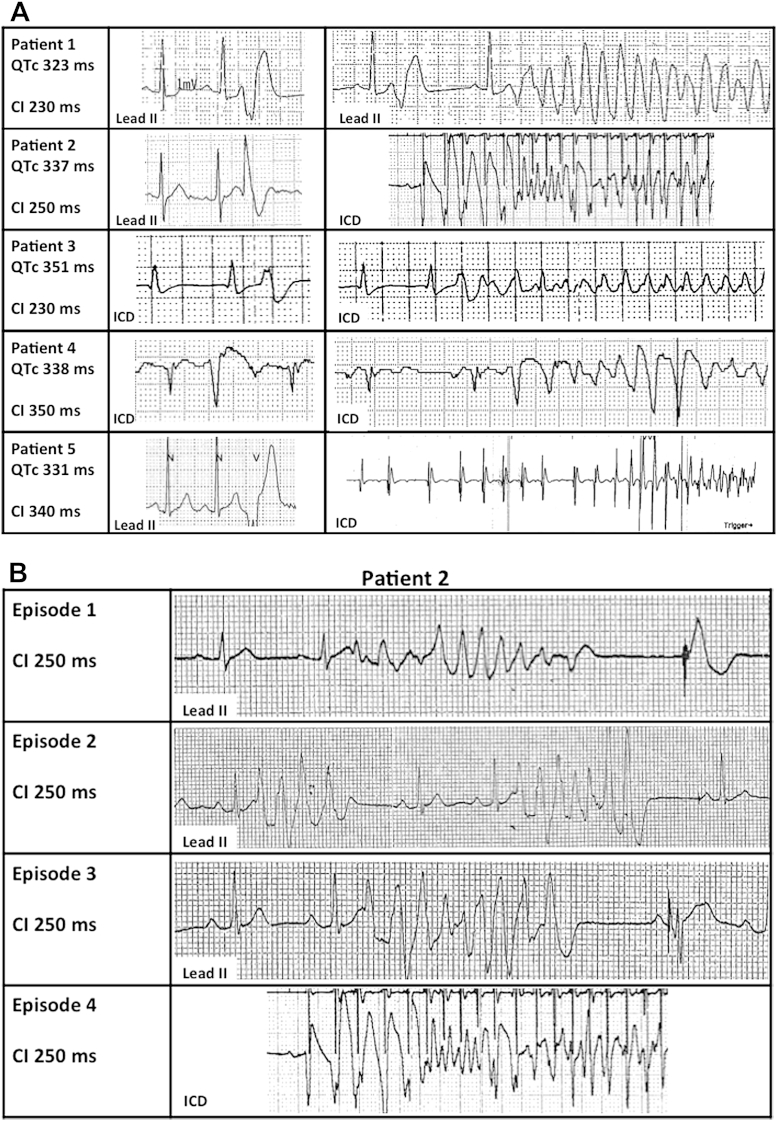
Onset of VF in Short QT Syndrome **(A)** Baseline QTc interval reported along with the coupling interval (CI) of the ventricular ectopic beat (VEB) initiating ventricular fibrillation (VF) **(column 1)**. Tracings of VEBs **(column 2)** with the same CI as the initiating beat of VF **(column 3)** are shown. VEBs occurred immediately before VF onset in Patients #1, #3, and #4. VF was initiated by VEBs with a short CI in Patients #1 to #3. **(B)** Patient #2 had multiple polymorphic nonsustained ventricular tachycardia episodes **(first 3 rows)** as a prelude to VF **(fourth row)** during an arrhythmic storm. Episodes are initiated by the same VEB (CI = 250 ms). ICD = implantable cardioverter-defibrillator.

We documented VF onset in 5 patients with an ICD (Fig. 5A). In 3 patients, the first beat of VF had a short coupling interval (CI) (basal QTc interval, 337 ± 14 ms; CI, 237 ± 12 ms; range, 230 to 250 ms), whereas the other 2 exhibited a long CI (basal QTc interval, 334 ± 6 ms; CI, 340 ms and 350 ms). No short-long-short onset sequences were observed.

In all patients, we found that isolated ventricular ectopic beats (VEBs) and/or the initiating beat of polymorphic nonsustained VT had the same CI of the beat precipitating VF (Fig. 5). Arrhythmias triggered by a short-coupled VEB presented the morphology of torsade de pointes and subsequently degenerated into VF. Arrhythmic events triggered by a long-coupled VEB exhibited disorganized electrical activity from the first beat.

**Figure 6:**
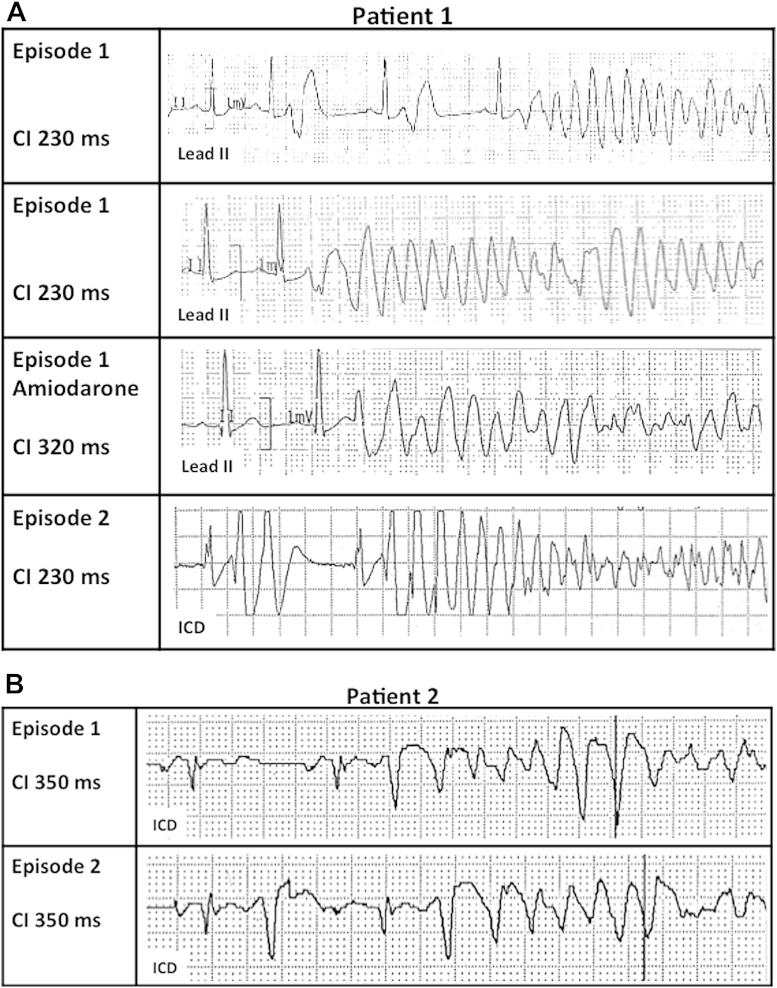
Multiple Episodes of VF in 2 Patients **(A)** Patient #1. Telemetry tracings **(rows 1 to 3)** from an arrhythmic storm and an ICD tracing **(row 4)** of an unrelated episode of VF. Arrhythmias (in the absence of amiodarone) were initiated by the same short-coupled (CI = 230 ms) ventricular extrasystolic beats. **(B)** Patient #2. Two successive episodes of VF within the same arrhythmic storm initiated by the same long-coupled ventricular extrasystolic beats (CI = 350 ms). Abbreviations as in Figure 5.

In 2 patients with multiple documented episodes of VF, the CI of the initiating beat was identical in all episodes in the same subject (Figs. 6A and 6B). In 1 patient, VF recurred during amiodarone infusion, precipitated by a different, longer coupled extrasystole (CI, 320 ms vs. 230 ms) (Fig. 6A).

## Discussion

In the present study, we report data from a cohort of 73 SQTS patients followed over a mean period of 5 years. The analysis of our population provides novel insights into the natural history, genetic profile, risk stratification schemes, and arrhythmogenesis of SQTS.

### Natural history of patients with SQTS

The paucity of SQTS patients reported in the published data is consistent with the nature of the genetic defects: gain-of-function mutations in proteins encoding voltage-dependent ion channels are limited by the fact that most amino acid changes are deleterious for these careful evolutionary-crafted structures. We should therefore anticipate that the management of SQTS patients will remain on the basis of small observational studies for quite some time.

Until now, the natural history of SQTS has been described by 1 multicenter study of 29 subjects (15). Our single-center cohort has twice the number of patients and allows a better definition of the age-related risk of death in SQTS. We calculated that in the 2006 study by Giustetto et al. (15), the annual rate of a first CA was 1.1% (10 of 29 patients had a first CA over a mean observation period of 32 years); likewise in our study group the annual rate of a first CA was 0.9% (20 of 73 patients had a first CA during a mean observation period of 31 years). Our data identify an interesting distribution of the occurrence of a first CA over time. We observed a peak incidence of CA in the first year of life (4% per year). Beyond these early months, we observed a quiescent phase encompassing adolescence, followed by an annual CA event rate of 1.3% between 20 and 40 years of age. Because SQTS and Brugada syndrome (BrS) belong to the spectrum of early repolarization syndromes (16), it is interesting to compare their natural histories.

Our data on BrS (17) showed that 15% of patients experience a first CA between birth and 44 years of age, corresponding to an annual event rate of 0.25%. In this study, we showed that by 40 years of age, as many as 40% of our SQTS patients experienced a first CA, with an annual event rate of 0.9%. It appears, therefore, that SQTS is more malignant than BrS, as also supported by the high rate of life-threatening arrhythmias in the first year of life.

### Sex differences

Similar to BrS, SQTS is an autosomal dominant disease, and it should be equally prevalent in male and female patients. However, when we establish the diagnosis on the basis of clinical parameters, excluding the contribution of genetic testing, the predominance of male patients is overwhelming (91%), suggesting that SQTS has, like BrS, a sex-dependent penetrance. However, the percent of patients who had life-threatening arrhythmias develop is not significantly different in male patients compared with female patients (30% vs. 17%, respectively; p = 0.49), suggesting that, in the clinics, we establish the diagnosis in more male patients than female patients, but we should not consider affected female patients at lower risk of CA.

### Familial and sporadic forms

In this study, we report novel data on the familial penetrance of SQTS. Familial clustering of the short QT phenotype was present in approximately half of SQTS probands, whereas the remaining were likely sporadic. The percent of familial disease is higher than that reported in BrS (28%) (18). In the familial cases, half of the first-degree relatives screened were clinically affected, consistent with the known autosomal dominant pattern of inheritance.

In line with the recent consensus document on genetic screening for channelopathies and cardiomyopathies (4), we confirmed that none of the genes related to SQTS identifies a disease-causing mutation in >5% of clinically affected probands. Interestingly, all the mutations found in our cohort showed a complete penetrance in carriers. Although the relatively small number of subjects limits our interpretation, this observation contrasts with that reported for *SCN5A* mutations in BrS (<50%) (17).

Mutation carriers exhibited a significantly shorter QTc interval (p = 0.002) compared with noncarriers, but this finding did not correlate with a different outcome, supporting the view that, at variance with long QT syndrome, the more severe electrocardiographic phenotype is not a prognostic indicator in SQTS (see the following) text.

### Risk stratification

To date, there are no data supporting the efficacy of pharmacological treatments to reduce the occurrence of life-threatening arrhythmias in SQTS (19), and implanting an ICD is the only treatment to prevent sudden death. It is therefore important to identify clinical markers to predict the risk of CA. Unfortunately, neither the presence of a very short QT interval nor the history of syncope alone identifies patients at higher risk of CA. Interestingly, analogous with what is seen in BrS (20), inducibility of VT/VF at PES is not an independent predictor of risk in SQTS.

In our population, the only predictor of malignant arrhythmias at follow-up is having experienced a first CA: these data strongly support ICD implantation in CA survivors, given that as many as two thirds of them had a recurrence at follow-up.

Considering the lack of individual predictors of risk in the asymptomatic population, we investigated whether the multiparametric risk score proposed by Gollob et al. 6, 7 could identify patients at risk of life-threatening events in our study group. Unfortunately, this hypothesis was not confirmed. When we applied the modified Gollob score to our study group, 5 of 8 patients who experienced CA had a score of ≤3, which corresponds to a predicted low probability of arrhythmic events. Overall, we urge caution regarding the use of this scoring system for risk stratification.

### Triggers for cardiac events

In our cohort, we observed that the circumstances in which CA occurred in patients who experienced multiple life-threatening events were reproducible in almost 80% of cases. The majority (83%) of CAs occurred under resting conditions or during sleep, whereas events related to effort or intense emotions were observed in a minority of patients (17%). In this respect, the arrhythmic triggers in SQTS appear more similar to BrS than to other channelopathies such as long QT syndrome or catecholaminergic polymorphic VT, in which arrhythmias are precipitated by adrenergic triggers.

### Electrophysiological characteristics of arrhythmias in SQTS

The presence of a short action potential duration, as well as an abbreviated repolarization, suggests that the R-on-T phenomenon may precipitate arrhythmogenesis in SQTS. Unexpectedly, however, we documented that either long- or short-coupled extrasystolic beats initiated VF in our patients. Although the mechanism for VF/VT initiated by long-coupled extrasystoles remains speculative, the arrhythmogenic mechanism for VF/VT elicited by short-coupled beats may be explained by the results of the in silico characterization of a *KCNJ2* gain-of-function mutation identified in one of our SQTS patients with an extremely short QT interval (14). In that study, we showed that the Purkinje network, because of its markedly abbreviated refractory period, creates a functional accessory pathway through which short-coupled extrasystoles could initiate reentry.

Interestingly, the mode of onset of VF observed in SQTS is similar to that seen in idiopathic VF, supporting the idea that some patients with idiopathic VF may have a forme fruste of SQTS (21).

### Study limitations

This study provides registry data and, although the authors clinically evaluated all patients, there has been no uniform protocol for clinical assessment and treatment. Moreover, ascertainment bias, which typically selects highly affected patients during the early characterization of disorders, is likely to be present. Furthermore, despite careful investigation into the nature of syncope, we cannot fully exclude that vagal episodes might have been present in some patients.

## Conclusions

Despite the limited number of patients with SQTS, the understanding of the clinical features of the disease is steadily progressing. The present study reports the largest series of SQTS patients prospectively followed and provides novel observations with a practical impact on clinical management. The evidence that there is an age dependency in the susceptibility to arrhythmias, with a peak in the occurrence of CA in the first year of life and a second peak between 20 and 40 years of age, is relevant. In fact, it points to the value of extending clinical evaluation to family members of SQTS probands and highlights the importance of electrocardiographic evaluation of newborns in SQTS families. Our data also show the presence of a strong male predominance in the manifestation of an abbreviated repolarization. However, we highlight the view that women should not be regarded as low-risk patients because female patients presenting with a short QT interval have a risk of experiencing CA that is similar to that of male patients.

The challenge for the management of patients with SQTS remains the paucity of risk stratification indexes. To date, no risk indicator has been identified. Our data show that the prognostic score proposed by Gollob et al. 6, 7 is not able to identify patients who experienced CA in our population. In this study, we showed that having survived a first occurrence of CA is a strong predictor of recurrences. This information is important because it supports the need to consider implanting an ICD for secondary prevention of CA even in young SQTS patients.

The most urgent challenge in SQTS is to identify indicators of a propensity toward CA among asymptomatic patients. Perhaps as larger series of patients with longer follow-up become available, it will be possible to provide evidence-based data for the management of asymptomatic SQTS subjects and tailor the use of the ICD to the higher risk subgroup.
