# Supplementary material for: Novel Insight Into the Natural History of Short QT Syndrome
Source: J Am Coll Cardiol. 2014 Apr 8;63(13):1300–8. doi: 10.1016/j.jacc.2013.09.078 (PMC3988978; doi:10.1016/j.jacc.2013.09.078)
Supplement: Online Figures 1–3 [file mmc1.docx]

**Online Appendix for the following *JACC* article**

**TITLE:** Novel insights in the natural history of Short QT Syndrome

**AUTHORS:**

Andrea Mazzanti, MD*, Ajita Kanthan, MBBS, PhD*, Nicola Monteforte, MD*, Mirella Memmi, PhD*, Raffaella Bloise, MD*, Valeria Novelli, PhD*, Carlotta Miceli, MS*, Sean O’Rourke, BS†, Gianluca Borio, MS *, Agnieszka Zienciuk-Krajka, MD‡, Antonio Curcio, MD, PhD*, Andreea Elena Surducan, MS§, Mario Colombo, MS§, Carlo Napolitano, MD, PhD*† and Silvia G Priori, MD, PhD*† ǁ

The first 2 Authors contributed equally to the manuscript.

The Authors thank Paola Baiardi, biostatistician, and Kevin Ng, MD, for their support in the revision of this manuscript.

The Authors also express their gratitude to the patients and to their referring Clinicians for the participation in the registry.

Online Figure 1


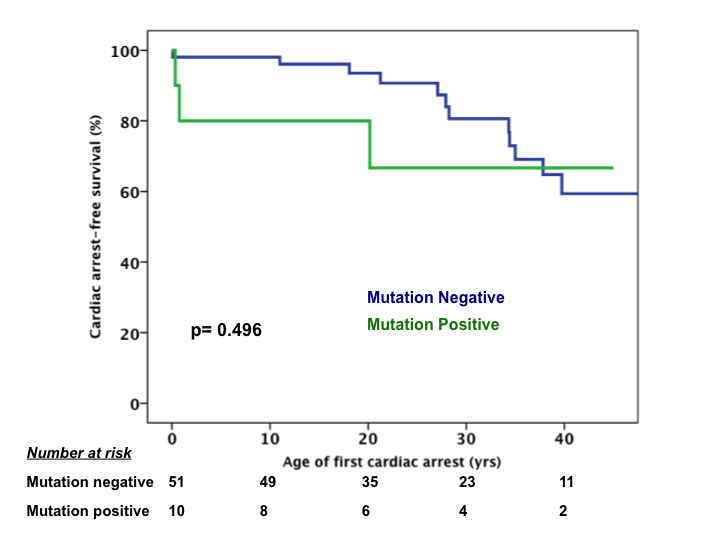


**Online Figure 1. Cardiac arrest-free survival by mutation status.** Kaplan-Meier analysis of patients that completed genetic screening on the genes KCNH2, KCNQ1, KCNJ2, CACNA1C, CACNB2: green line represents mutation carriers (n=10); blue line represents mutation non-carriers (n=51). There was no significant difference between both groups (p=0.496).

Online Figure 2


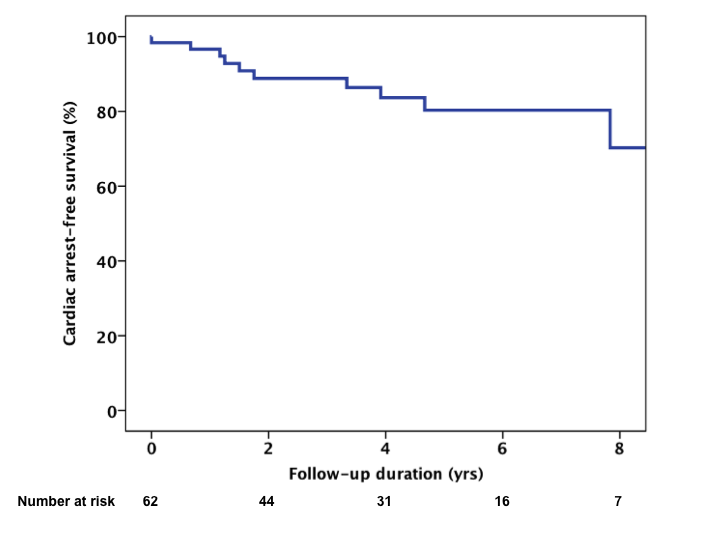


**Online Figure 2. Cardiac arrest-free survival at follow-up.** Kaplan-Meier analysis for 62 patients with follow-up (mean follow-up duration 60±41 months; median 56 [IQR 43] months).

Online Figure 3


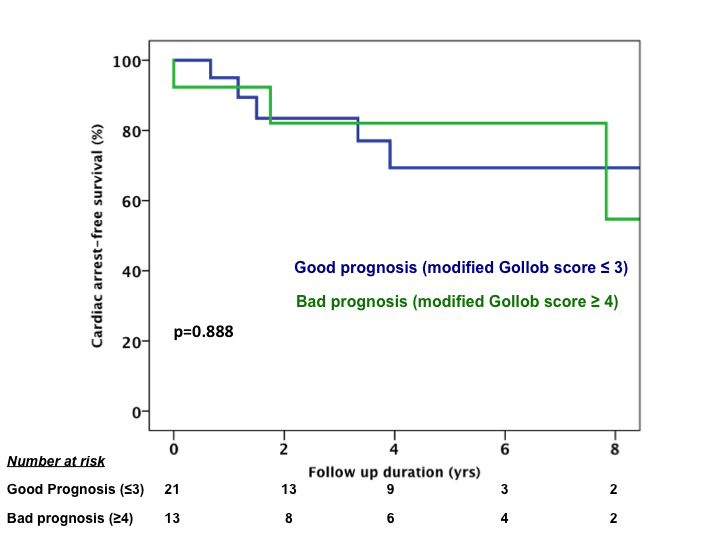


**Online Figure 3. Cardiac arrest-free survival at follow-up by modified Gollob score.** Kaplan-Meier analysis: blue and green lines respectively represent patients at low (score ≤3, n=21) and high (score ≥4, n=13) risk of cardiac arrest. There was no significant difference between both groups (p=0.888).
